# Synthesis Metakaolin-Based Geopolymer Incorporated with SiC Sludge Using Design of Experiment Method

**DOI:** 10.3390/polym14163395

**Published:** 2022-08-19

**Authors:** Kang-Wei Lo, Wei-Ting Lin, Ya-Wen Lin, Ta-Wui Cheng, Kae-Long Lin

**Affiliations:** 1 Institute of Mineral Resources Engineering, National Taipei University of Technology, Taipei City 106, Taiwan; 2 Graduate Institute of Engineering Technology, National Taipei University of Technology, Taipei City 106, Taiwan; 3 Department of Environmental Engineering, National Ilan University, No.1, Sec. 1, Shennong Rd., I-Lan 260, Taiwan

**Keywords:** SiC sludge, design of experiment, factor parameter, interaction, multivariate regression model

## Abstract

This study uses metakaolin, sodium hydroxide, sodium metasilicate, and water content as the reaction variables in the application of the design of experiment (DOE) method. At the same time, the various component factors and their interactions were analyzed to understand how these factors affect the mechanical properties of a metakaolin-based geopolymer incorporated with SiC sludge (SCSGPs). The results of the statistical analysis showed that the compressive strength of SCSGPs was affected by the Na/Si molar ratio (NSR) (*p*-level = 0.000 <0.05), the Na/Al molar ratio (NAR) (*p*-level= 0.000 <0.05), and the interaction between the dissolution rate of Si (DRA). Within the design scope of this study, the maximum compressive strength of SCSGPs and the coefficients of the NSR, the NAR, and the DRA of SCSGPs was evaluated. The multiple regression analysis and the tested coefficient of r^2^ were also studied. The multiple regression analysis models provide an effective reference for the application of SCSGPs.

## 1. Introduction

In 1978, Joseph Davidovits created the terminology “geopolymer” to describe inorganic, amorphous, and semicrystalline aluminosilicates [1]. Geopolymerization involves the chemical reaction of aluminosilicate with silicates under highly alkaline conditions [2] to generate polymeric Si–O–Al–O bonds [3]. Metakaolin (MK) is a commonly used aluminosilicate material. In alkaline media, it dissolves to form single Al(OH)_4_^−^ and Si(OH)_4_^−^ species, which undergo polymerization [4]. Alkali metal salts and hydroxides are dissolved to form silicon dioxides and alumina for catalytic condensation reactions [4]. According to some reports, geopolymers exhibit excellent performance and are fire resistant [5,6]. The study and exploitation of various kinds of geopolymers have demonstrated their use as fireproof materials [7], cement and concrete [8,9], thermal insulation materials [10], refractory materials [11], and high-tech composite materials [12]. Many factors affect mechanical behavior following powder particle dissolution, polymerization, and hardening reaction processes [13]. Regarding the mechanical properties, compressive strength was the focus. Only a few studies have applied engineering to exploit other mechanical factors.

Sodium metasilicate (Na_2_SiO_3_) can provide sufficient silicon ions during the polymerization process to promote the activation precursor of geopolymer materials and improve the mechanical properties [14], and Xu and Van Deventer (2003) discovered a simple correlation between the sodium silicate solution concentration and the compressive strength of the prepared geopolymer [15]. Many factors influence the structural properties of geopolymers. One technique to find influencing factors from geopolymers was the design of experiments [16,17]. Factorial design and mixture design are statistical techniques for the design of experiments (DOEs) aiming to evaluate the linear, nonlinear, and interaction effects of several factors (independent variables) on a measurable property or response (dependent variable) [16,17]. The compressive strength was found to be affected by the microstructure and the corresponding composition contents [18]. The mechanical properties and microstructure were shown to be controlled by three factors, including the Si/Al ratio [18], the Na/Al ratio [19], and the H_2_O/Na ratio [20]. Natassia (2021) evaluated the use of rice husk ash and aluminum anodizing sludge as aluminosilicate sources to obtain geopolymers in a mixture of sodium silicate and sodium hydroxide used as alkaline solutions. The analysis of variance (ANOVA) results indicated that the aluminum sludge is the factor that most affects the apparent density, diametral shrinkage, tensile strength, and deformation at rupture. The higher the sodium silicate + sodium hydroxide solution content, the lower the apparent density, shrinkage, and deformation at rupture [21]. João (2021) evaluated the valorization of clay ceramic waste as a raw material for the synthesis of geopolymers. Clay ceramic waste, sodium hydroxide, and sodium silicate were the independent variables of a mixture design used to define the geopolymeric system. The results indicated that the amount of silica (SiO_2_ = 69.8) and alumina (Al_2_O_3_ = 17.3) on the clay ceramic waste results in a Si/Al ratio of 4.0, which is adequate for geopolymerization, with adequate strength at 28 days of age [22].

Furthermore, Tailby and Mackenzie (2010) analyzed one combination that influenced the mechanical strength and microstructure of a curing polymer, and geopolymerization occurred easily in a highly alkaline environment [23]. However, the excess Ca and OH^−^ ions in the reaction process might have caused the formation of Ca(OH)_2_, hindering geopolymer formation and resulting in an incomplete geopolymer structure [19]. Rovnanik (2010) examined the mechanical properties of a geopolymer by analyzing the work factor, and his results revealed an increase in the early strength of the geopolymer at high curing temperatures [24]. A definite relationship between the key bond and structure type of a geopolymer and its strength, which required an interpretation of the relationships between the polymerization, structural properties, and mechanical properties, was not determined [24]. Xiao et al. (2020) indicated that due to the synergy between geopolymers and waste material, the strength performance improved significantly after a curing time of 14 days, which was attributed to the reaction between waste glass and class C fly ash [25]. Sourav (2021) examined the effect of the ratio of sodium silicate to sodium hydroxide and Na_2_O% with different alkali proportions on the hardened properties. The results revealed that the optimum compressive strength of 20.7 MPa is achieved at a sodium silicate to sodium hydroxide of 1.54 and Na_2_O% of 12.5% [26]. Iman (2021) examined sandy soil stabilized using a geopolymer based on copper mine tailing dam sediments with different content (10–20%) in different concentrations of potassium hydroxide (1–10 M). The results indicated that a geopolymer based on copper mine tailing dam sediments can be utilized to efficiently improve the compressive strength of soil without damaging the environment. The inclusion of silica fume in the mixtures considerably improved the strength and microstructural density of the specimens [27]. The goals of the Waste Electrical and Electronic Equipment directive are to prevent the inappropriate disposal of electrical and electronic equipment waste, reuse and recycle waste, and reduce e-waste deposition in landfills. This directive highlights the need to address the SiC sludge (SCS) problem. To our knowledge, the DOE approach has not been used to optimize the strength of a geopolymer obtained from SCS and MK [22,23,24,25,26,27]. Based on previous studies [22,23], optimization of design factors for a metakaolin-based geopolymer incorporated with SiC sludge (SCSGPs) was proposed using the DOE approach in this work. Mechanical properties were analyzed to determine the significant component factors based on the independent variable in the statistical analysis. The influences of the interactions between different component factors were considered, and it was expected that scientific data and a standardized design process for SCS admixtures would be provided for the production of suitable geopolymers.

## 2. Materials and Methods

### 2.1. Design of Experiments (DOEs)

**Figure 1:**
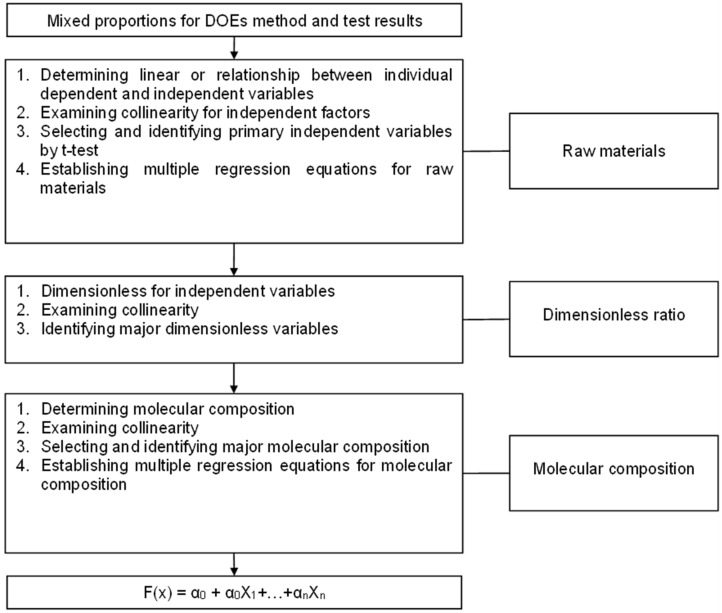
Flowchart of statistical analysis.

To determine the influence of componential factors and the effect of the associated interaction among these components on subsequent mechanical characteristics, this study adopts the DOEs to prepare SCSGPs. Four factors, MK, sodium hydroxide (NaOH), Na_2_SiO_3_ and the water content, were employed as reaction variables in the application of the DOEs. The individual component factors and the interactions between them were analyzed to determine the effects on the mechanical properties of SCSGPs. Previous research treated these factors (MK, NaOH, Na_2_SiO_3_, and the water content) as important components influencing the compressive strength of SCSGPs [11,18,19,20,28]. Each factor included three levels of the component content: high, medial, and low. Si/Na = 0.5 − 2.5, S/L= 0.2 − 1.2 and OH^−^ (M) = 0.67 − 12.46. Therefore, the mixed combination of SCSGPs was designed into 9 groups. Table 1 lists the 3^4^ (L_9_) orthogonal arrays of the four research factors and the related design mixing ratios. When preparing SCSGPs samples using these mixture ratios (Table 1), all mixing methods and curing processes were fixed. According to the experimental results, the F-value and p-value (p-value) were calculated to study the individual influence of each design parameter. According to the experimental design method, there are L_9_ (3^4^) orthogonal arrays for factor setting and sample preparation, SPSS Statistics 27.0 software for ANOVA and univariate, multivariate, etc., to determine the impact of different factors. Additives have a significant impact on compressive strength and clarify the interaction between different factors. Figure 1 shows the flowchart of multiple statistical analyses and multiple regression analyses.

### 2.2. Application of Experimental Design Method in Optimizing Design Parameters

More variables used in the production of geopolymers (including inconsistent raw materials, alkali activators, and curing) have led to complexity in the design of their mixtures. Related research reports point out that the properties of geopolymers are affected mainly by factors such as raw materials, alkali activator types, and concentrations [14,15,16,17,18]. Previous studies have shown that the silicate ratio is the main influencing factor for the formation and strength development of geopolymers microstructures [14,15,16,17,18]. The statistical factor model method can provide time efficiency for geopolymers design and has a balance between design parameters. In this study, on the preparation of SCSGPs, experimental design methods were used to obtain experimental parameter settings. The four main control factors, the SiO_2_/Na_2_O ratio (Si/Na), the SiO_2_/Al_2_O_3_ ratio (Si/Al), the solid/liquid ratio (S/L), MK, and SCS, were selected based on the results of Section 2.1, and the mixing combinations of the SCSGPs led to thirty-five experiments that were run. Table 2 lists the design mixing ratios related to the four research factors. Multivariate regression analysis and ANOVA were employed to determine the significance of the influences of the different additive factors and to clarify the interactions between the different component factors. Finally, a multivariate regression model was established based on the experimental results. In addition, microstructure analysis and macroscopic property tests were used to explore and understand the reaction mechanism of silicon carbide sludge on inorganic polymers and to verify its influence on the macroscopic and microscopic properties of the inorganic polymerization reaction mechanism.

### 2.3. Materials and Sample Preparation

The SCSGP in this study was made of kaolin, SCS and alkaline solution. MK was generated from kaolin by calcination at 650 °C in the air for 3 h. The SCS used in this study was collected from the blue light-emitting diode (LED) manufacturing plant in Taiwan. Raw SCS was milled using chrome steel balls and passed through a 0.074 mm sieve. After crushing in a ball mill, the fineness of the SCS powder was controlled to 300–400 m^2^/kg. The composition of SCS consisted of 75.40% SiO_2_, 0.80% Al_2_O_3_ and 23.00% SiC. On the other hand, MK consisted of 51.80% SiO_2_, 43.00% Al_2_O_3_ and 1.30% Fe_2_O_3_. Reagent-grade NaOH was added to deionized water and allowed to release heat for 24 h. Alkali solution, with required Si/Na (as shown in Table 1), was prepared by using sodium metasilicate and NaOH solution. While the replacement levels of SCS were 0%, 10%, 20%, 30% and 40% of MK content in weight. After the mixture was stirred at a medium speed for another 5 min, the samples were subsequently cast into 25.4 mm × 25.4 mm × 25.4 mm cubic plastic molds. Air was removed from the samples using a shaker, and the samples were subsequently cured at a constant temperature of 30 ± 2 °C and constant humidity for 1 day. The samples were removed from the molds and then further cured under the same conditions for 28 days. The physical properties and the compressive strength of the metakaolin-based geopolymer incorporated with SiC sludge were tested according to ASTM C109 [29]. Three specimens underwent used in for the compressive strength tests while the microstructure of the fourth was determined. The average strength of the three specimens is presented. The coefficient of variation of these results was less than 10%.

### 2.4. Test Items and Methods

The geopolymer analysis was classified according to the factor parameters, mechanical properties, and microstructure in this study, including multivariate regression analysis of mechanical properties. The factor parameters contain the concentrations of Si, Al, and Na ions. According to the mix proportions in Table 1 and Table 2, the ion concentrations of the MK, SCS, and alkaline solution were measured in separate tests. First, the MK, SCS, and alkaline solution were stirred for 60 min. Subsequently, 3.8 L of deionized water was added to the mixture and the suspension was stirred for another 30 s. After filtration, the dissolved Si, Al, and Na concentrations in the solution were measured by atomic absorption spectroscopy (AAS) [30]. The mechanical properties of the SCSGPs were tested according to ASTM C109 [29]. The mix proportions of the SCSGPs are listed in Table 1 and Table 2. Take three samples for each test and get the average for the results. In this study, SPSS Statistics 27.0 software was used to perform variance analysis and regression analysis of single variables and multiple variables, etc., to determine the significant factors of different additives, and to clarify the interaction between different factors. The microstructural properties were analyzed by scanning electron microscopy (SEM). SEM images were obtained using a Hitachi S-3500 N to show the SCSGP microstructure. These scientific data are expected to facilitate the application of SCSGPs and the standardization of the design process of SCSGPs.

## 3. Results and Discussion

### 3.1. Factor Parameter and Physical Property Test

The compressive strength results for the metakaolin-based geopolymer and the mix proportions are listed in Table 1. The compressive strengths for the mix proportions were 0.47–49.44 MPa in the DOEs range. The highest strength was 105 times the lowest strength, but no strength value was measured for the mix proportion of No. 9. because the high concentration of sodium metasilicate when Si/Na exceeds 2.0 will reduce the reactivity of geopolymerization, and the system does not have enough alkaline solution in the case of a higher S/L ratio, so that the geopolymer cannot be formed. Therefore, the compressive strength of No. 9 was not tested. The experimental results showed that the strength values were distributed over a wide range, which helped identify the influence order of the strength factors of the metakaolin-based geopolymer. The compressive strength decreased as Si/Na increased from 1.6 to 2.0. Cho et al. (2017) indicated that when the Na_2_O content increases from 4.0% to 6.0%, the strength after curing 28 days increases to 348%; however, when the Na_2_O content increases from 6.0% to 8.0% and 8% to 10%, the rate of strength development drops to 181% and 115%, respectively [31]. This result is consistent with the results of Cho et al. (2017).

### 3.2. One-Way ANOVA

The aforementioned single-variable regression analysis results show that non-simple linear regression can effectively describe the properties of the metakaolin-based geopolymer. These univariate regression analysis results indicated that interactions between the four-factor parameters, the Si/Na, S/L, and Si/Al ratios and OH^−^ (M), affected the compressive strength of the SCSGPs. Therefore, in this section, the F-values and *p* values of the alkaline solution (Si/Na and OH^−^ (M)), the S/L ratio, and the Si/Al ratio were compared by one-way ANOVA to clarify the strengths of the influence of the different variables, which would be beneficial for optimizing the application of design factors to SCSGPs.

Table 3 shows the one-way ANOVA results for the four-factor parameters of the compressive strength of the SCSGPs. The results showed that the sum of squares reached 2.517 for Si/Na, and the F-value of 18.776 exceeded the critical value of 4.49, which treated the result at the 95% confidence interval. Therefore, Si/Na was determined to be a significant factor affecting the compressive strength. The sum of squares of OH^−^ (M) reached 114.379, and the F-value of 13.727 exceeded the critical value of 4.49; thus, OH^−^ (M) was also determined to be a significant factor influencing the compressive strength. The sum of squares of the S/L and Si/Al ratios were 0.7 and 0.135, respectively. Moreover, the F-values of the S/L and Si/Al ratios were 19.737 and 19.381, respectively, which also exceeded the critical value of 4.49; therefore, the S/L and Si/Al ratios were also found to be significant factors affecting the compressive strength. The *p* value showed the degree of effectiveness of each term. Each factor with a *p* value less than 0.05 is considered to be effective parameter. The calculations of the *p* value at the 95% confidence interval were analyzed because the influence levels could not be differentiated by the F-values. The *p* value of the Si/Na, S/L, and Si/Al ratios and OH^−^ (M) was 5.14 × 10^−4^, 4.09 × 10^−4^, 4.45 × 10^−4^, and 1.92 × 10^−3^, respectively, and the influence levels of the factors were the S/L ratio > the Si/Al ratio > the Si/Na ratio > OH^−^ (M). The influence levels of the S/L and Si/Al ratios were higher than the influence levels of the Si/Na ratio and OH^−^ (M), which was consistent with the results of Section 3.1.

### 3.3. Application of Optimization Design Factors by the Multivariate Statistical Analysis

Table 2 shows the compressive strength results of the SCSGPs and the mix proportions. The compressive strengths of the mix proportions were 3.52–66.08 MPa in the design range. In this study, multivariate statistical analysis was employed to determine the crucial factor influencing the mechanical parameters of the SCSGPs. A multiple regression analysis established the relationship between the factor parameters. It was important to discriminate between a linear and nonlinear relationship between the independent and dependent variables before the multivariate statistical analysis. This study also examined the collinearity of the independent variables, and one factor was selected based on collinearity to avoid having to use the significant relationship of the independent variable. Based on the residual sum of squares of the multivariate statistical analysis, the major *t* variable was selected for independent-samples *t*-tests, and the other factors were increased or decreased to further test the selected variable to identify significant relationships with the independent variable and interactions between the factors. Finally, the crucial factor relationship with the mechanical properties of the SCSGPs was analyzed, and an MRM and multiple regression equation were established based on the experimental results.

#### 3.3.1. Raw Material Analysis

**Figure 2:**
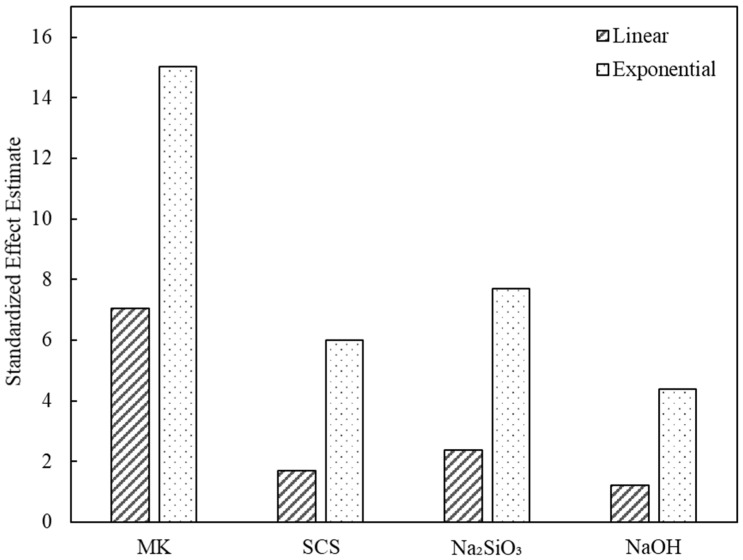
Statistical analysis results of standardized effects between compressive strength and raw materials of the SCSGPs.

Figure 2 shows the statistical analysis results for the standardized effects of the raw materials of the SCSGPs on its compressive strength. All the raw materials (i.e., MK, SCS, Na_2_SiO_3,_ and NaOH) were used to describe the exponential relationship with the compressive strength, and all the variance inflation factor values were below 10. Table 4 lists the results of the multiple regression analysis of the compressive strength and raw materials. The results of the t-tests showed that MK was the most significant factor in terms of compressive strength, followed by Na_2_SiO_3_. According to the interaction factors, the strongest positive relationships were observed between MK and NaOH and between NaOH and Na_2_SiO_3_. The highly soluble silica from Na_2_SiO_3_ incorporates leached silica and alumina from the raw material into an N-A-S-H gel. Metakaolin solubility is expected due to the typical presence of a substantial amorphous aluminosilicate phase, which is generally easily dissolved in sodium hydroxide solutions.

#### 3.3.2. Dimensionless Ratio Analysis

After separating the raw materials, multiple regression analysis was performed again to determine the relationship between the compressive strength of the SCSGPs and the dimensionless ratios (Si/Na, S/L, OH^−^ and OH^−^/water), as shown in Table 5. According to the results of the *t*-tests (Table 5), OH^−^/water (OW) had the most influence on the compressive strength of the SCSGPs, followed by SL and then SN. Therefore, the significant factors influencing the compressive strength of the SCSGP were inferred to be OH^−^, SiO_2_, Na_2_O, and H_2_O. Under highly alkaline conditions, the rapidly dissolving aluminosilicate released [SiO_4_]^−^ and [AlO_4_]^−^ into the liquid, and each tetrahedron shared its oxygen atoms with the precursor substance of the polymer [32,33] to generate polymeric Si–O–Al–O bonds [3,33].

However, the variance inflation factor results showed that OH^−^ (mol) and Na_2_O (mol) exhibited high collinearity when OH^−^ was treated as an overall reaction concentration, not overall alkalinity. Therefore, OH^−^ (mol) and subsequently the Na/Si molar ratio (NSR), the Na/Al molar ratio (NAR), the dissolution rate of Si (DRS), and the dissolution rate of Al (DRA) were selected to calculate the molar concentrations of the factors in the reaction process for the multiple regression analysis to determine their influences on the compressive strength of the SCSGPs. Table 6 lists the results of the multiple regression analysis of the compressive strength and molecular composite ions (e.g., the NSR, the NAR, the DRS, the DRA, and OH^−^), which also revealed the interactions between significant factors. The results of the multiple regression analysis found strong negative interactions between the NSR and the DRA as well as between the NAR and the DRA, suggesting that the NSR and the NAR were the most significant factors, followed by the DRA. The test coefficient of r^2^ was 0.869, indicating its good fit of the quadratic model to the data. This result showed that these three factors were sufficient for describing the compressive strength of the SCSGPs, and the multiple regression equation was as follows.

(1) Compressive Strength (MPa)=0.550(NSR)−0.495(NAR)−0.383(DAR)−0.211(NSR)(DRA)

**Figure 3:**
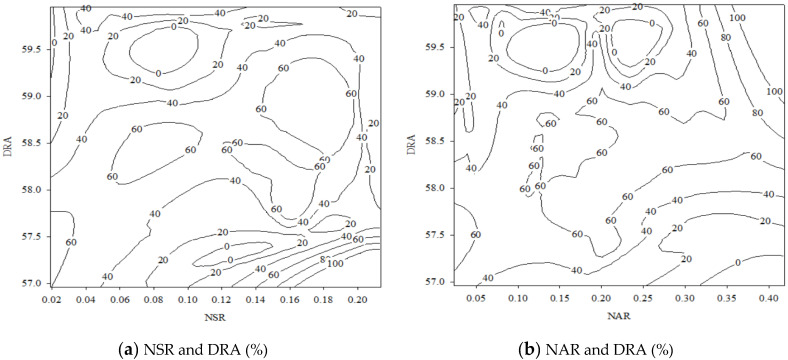
Relationship between compressive strength and the first three significant factors of the SCSGPs, i.e., (**a**) the NSR and the DRA (%); (**b**) the NAR and the DRA (%).

Figure 3 illustrates the MRM relationship between the compressive strength and the first three significant factors, i.e., the NSR and the DRA, in addition to the NAR and the DRA. In the design range in this study, the highest compressive strength of the SCSGPs was 66.08 MPa, and the NSR, NAR, and DRA factors of the SCSGPs were 0.15, 0.22, and 58.24, respectively. The study on MK-slag-clinker blends by Fernández-Jiménez et al. [34] suggested that Na^+^ favored coagulation/precipitation. The sample made with Na^+^ exhibited higher compressive strength of the SCSGPs.

### 3.4. Microstructural Analysis

**Figure 4:**
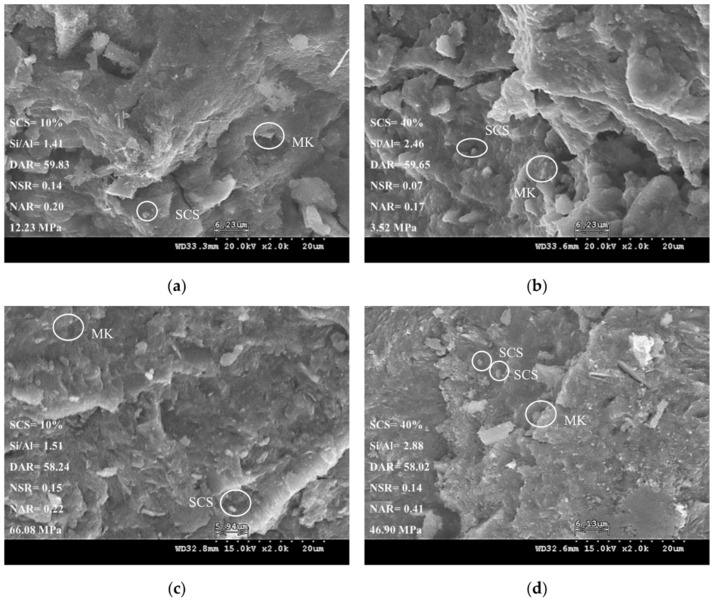
Micrographs of SCSGPs with different mix design parameters and compressive strength (SCS = 10% and 40%, the NSR = 0.07–0.15, the NAR = 0.17–0.41, the DAR = 58.02–59.83, and compressive strength (MPa) = 3.52–66.08), i.e., (**a**) SCS = 10% and the Si/Al = 1.41, (**b**) SCS = 40% and the Si/Al = 2.46, (**c**) SCS = 10% and the Si/Al = 1.51, (**d**) SCS = 40% and the Si/Al = 2.88.

The microstructure of the SCSGPs was changed due to the degree of polymerization. This change is affected by the complex interaction between the aluminosilicate raw material, curing conditions, the type of alkali activator, the ratio of alkali activator to the raw material, and the evolution of the chemical environment during the geopolymerization reaction. The SEM image shown in Figure 4 included four kinds of SCSGPs, i.e., those with mixed proportions of Nos. 12, 15, 27, and 30. The SCSGPs had a different microstructure at a DRA of approximately 59 (Nos. 12 and 15) than at a DRA of approximately 58 (Nos. 27 and 30). When the DRA was approximately 59, unreacted particles of MK and SCS existed in the microstructure, whereas at a DRA of approximately 58, the microstructure consisted of evenly distributed dense particles. The results showed that the compressive strength of the SCSGPs with a DRA of approximately 59 was lower than the compressive strength of the SCSGPs with a DRA of approximately 58. Because the Si–O–Si bond contents increased and the bond strength of the Si–O–Si bonds was higher than the bond strength of the Si–O–Al bonds, the microstructure of the SCSGPs was improved. These research findings were similar to the research findings of previous studies [35,36]. Modification of the other factor parameters, e.g., increasing the Si/Al ratio from 1.41 to 2.88, caused the structural form to change. Fletcher et al. (2005) and He et al. (2016) reported that when the Si/Al ratio exceeded 2.5, the amorphous products of the geopolymer decreased with increasing SiO_2_ content, and the shape changed easily [35,36].

The microstructures of the SCSGPs varied slightly when the DAR values were similar. The SCSGPs with similar DAR values had different NSR and NAR values, which led to the large difference in their microstructures. Theoretically, the Si/Na and Na/Al ratios can balance the charge between alkali metal cations and tetrahedral coordinated silicon and aluminum, as described in the literature [37,38,39,40]. In addition, a higher SCS content in the SCSGPs might have inhibited synergistic reaction effects [21,41], causes aluminosilicate to precipitate and cover the surface of MK and SCS particles, thereby reducing the dissolution reaction activity, forming a loose structure of macropores and reducing its compressive strength development [21,41].

## 4. Conclusions

In this study, the DOE method was used to perform a statistical analysis of experimental data to study the factors that affect the mechanical properties of SCSGPs. The result of the *t*-test shows that in terms of compressive strength, MK was the most important factor, followed by Na_2_SiO_3_. The *p* value of the Si/Na, S/L, and Si/Al ratios and OH^−^ (M) was 5.14 × 10^−4^, 4.09 × 10^−4^, 4.45 × 10^−4^, and 1.92 × 10^−3^ respectively. The influence level of factors was S/L > Si/Al > Si/Na > OH^−^. The influence level of the S/L and Si/Al ratios is higher than that of the Si/Na ratio and OH^−^. Using multivariate statistical analysis methods, the relationship between the mechanical properties of geopolymers and related parameters was predicted. After adjusting the multivariate test coefficients, the r^2^ value was greater than 0.869. The statistical analysis shows that the compressive strength of SCSGPs was affected by the interaction between the NSR and the DRA as well as between the NAR and the DRA, indicating that the NSR and the NAR were the most important factors, followed by the DRA. Within the design range of this study, the highest compressive strength of SCSGPs was 66.08 MPa, and the coefficients of the NSR, the NAR, and the DRA of SCSGP were 0.15, 0.22, and 58.24, respectively. The analysis results of the multiple regression analysis models provide an effective reference for the application of a metakaolin-based geopolymer incorporated with SiC sludge, and the required strength level can be achieved without tedious mixing formulas.

## Figures and Tables

**Table 1:** Mix proportions and compressive strength of the experimental design for studied factors. (The designed mix proportions according to the L_9_ 3^4^ orthogonal arrays.)

No.	Na_2_SiO_3_	MK	Water	NaOH	Si/Na	S/L	OW	OH^−^ (M)	Compressive Strength (MPa)
1	Low	Low	Low	Low	0.50	0.20	0.02	0.67	2.04 ± 1.56
2	Low	Medium	Medium	Medium	0.80	0.80	0.03	3.05	43.40 ± 2.30
3	Low	High	High	High	1.00	1.20	0.05	5.40	30.82 ± 3.21
4	Medium	Low	Medium	High	1.50	0.40	0.03	9.67	13.80 ± 1.82
5	Medium	Medium	High	Low	1.60	1.00	0.04	1.60	49.44 ± 14.04
6	Medium	High	Low	Medium	1.60	1.20	0.02	4.52	22.55 ± 2.06
7	High	Low	High	Medium	2.00	0.60	0.04	4.96	40.08 ± 6.01
8	High	Medium	Low	High	2.50	1.00	0.02	12.46	0.47 ± 0.07
9	High	High	Medium	Low	3.00	2.20	0.03	2.84	N/A

Note: MK: metakaolin; S/L: solid/liquid; OW: OH^−^/water; N/A: not tested.

**Table 2:** Mix design and compressive strength of the thirty-five (35) runs for SCSGPs.

No.	Si/Na	S/L	MK (%)	SCS (%)	Strength (MPa)
1	0.8	1.0	100	0	50.07 ± 3.25
2	0.8	1.0	90	10	47.16 ± 5.13
3	0.8	1.0	80	20	48.43 ± 6.77
4	0.8	1.0	70	30	37.47 ± 1.60
5	0.8	1.0	60	40	37.00 ± 3.37
6	1.2	1.0	100	0	57.67 ± 7.57
7	1.2	1.0	90	10	63.64 ± 4.20
8	1.2	1.0	80	20	63.05 ± 9.81
9	1.2	1.0	70	30	59.24 ± 8.79
10	1.2	1.0	60	40	54.47 ± 3.36
11	1.6	0.4	100	0	17.18 ± 0.84
12	1.6	0.4	90	10	12.23 ± 1.09
13	1.6	0.4	80	20	9.71 ± 0.47
14	1.6	0.4	70	30	7.28 ± 0.62
15	1.6	0.4	60	40	3.52 ± 0.22
16	1.6	0.6	100	0	43.08 ± 9.02
17	1.6	0.6	90	10	36.26 ± 3.58
18	1.6	0.6	80	20	28.43 ± 4.83
19	1.6	0.6	70	30	24.61 ± 1.72
20	1.6	0.6	60	40	15.53 ± 1.72
21	1.6	0.8	100	0	61.76 ± 4.40
22	1.6	0.8	90	10	59.18 ± 5.50
23	1.6	0.8	80	20	57.71 ± 9.43
24	1.6	0.8	70	30	51.00 ± 2.81
25	1.6	0.8	60	40	26.88 ± 1.92
26	1.6	1.0	100	0	64.37 ± 7.71
27	1.6	1.0	90	10	66.08 ± 9.13
28	1.6	1.0	80	20	64.59 ± 6.77
29	1.6	1.0	70	30	60.34 ± 8.86
30	1.6	1.0	60	40	46.90 ± 2.94
31	2.0	1.0	100	0	61.51 ± 3.61
32	2.0	1.0	90	10	54.43 ± 5.82
33	2.0	1.0	80	20	31.09 ± 5.11
34	2.0	1.0	70	30	17.35 ± 1.83
35	2.0	1.0	60	40	14.05 ± 3.05

**Table 3:** Results of one-way ANOVA for compressive strength and factor parameter of the SCSGPs.

Source	Sum of Squares	Degree of Freedom	Mean Square	F	*p*-Value
Si/Na	2.517	7	0.36	18.776	5.14 × 10^-4^
Error	0.005	1	0.005		
Total	2.522	8			
Solid/Liquid	0.7	7	0.1	19.737	4.09 × 10^-4^
Error	0.02	1	0.02		
Total	0.72	8			
Si/Al	0.135	7	0.019	19.381	4.45 × 10^-4^
Error	0.054	1	0.054		
Total	0.189	8			
OH^−^ (M)	114.379	7	16.34	13.727	1.92 × 10^−^^3^
Error	2.247	1	2.247		
Total	116.626	8			

**Table 4:** Multiple regression analysis for compressive strength and raw materials of the SCSGPs (r^2^ = 0.984).

Factor	Coefficient	Standard Error	*t* (35)	*p*-Value (<0.05)
(1) MK	29.960	1.996	15.013	0.000
(2) SCS	−25.540	4.186	−6.101	0.000
(3) Na_2_SiO_3_	39.785	5.180	7.681	0.000
(4) NaOH	−15.365	3.512	−4.375	0.000
(1) by (2)	55.500	4.537	12.233	0.000
(1) by (3)	−9.825	4.743	−2.072	0.045
(1) by (4)	45.325	3.336	13.588	0.000
(2) by (3)	−65.325	3.824	−17.085	0.000
(2) by (4)	−10.175	2.624	−3.878	0.000
(3) by (4)	55.150	3.637	15.163	0.000

**Table 5:** Multiple regression analysis for compressive strength and dimensionless ratio of the SCSGPs (r^2^ = 0.826).

Factor	Coefficient	Standard Error	*t* (35)	*p*-Value (<0.05)
Si/Na	−42.540	3.142	−13.538	0.000
S/L	−43.190	3.106	−13.905	0.000
OW	−43.896	3.131	−14.020	0.000

**Table 6:** Multiple regression analysis for compressive strength and molecular composite ion of SCSGPs (r^2^ = 0.869).

Factor	Effect	Std. Err.	*t* (40)	*p*-Level (<0.05)
(1) NSR	−43.918	3.129	−14.038	0.000
(2) NAR	−43.845	3.130	−14.008	0.000
(3) DRS	−1.487	2.329	−0.639	0.527
(4) DRA	14.651	3.187	4.597	0.000
(5) OH^−^	−39.612	3.159	−12.539	0.000

## Data Availability

The data presented in this study are available on request from the corresponding author.
